# Volumetric reduction of brain metastases after stereotactic radiotherapy: Prognostic factors and effect on local control

**DOI:** 10.1002/cam4.4809

**Published:** 2022-05-10

**Authors:** Naoyuki Kanayama, Toshiki Ikawa, Shingo Ohira, Takero Hirata, Masahiro Morimoto, Kazuhiko Ogawa, Teruki Teshima, Koji Konishi

**Affiliations:** ^1^ Department of Radiation Oncology, Osaka International Center Institute Osaka Japan; ^2^ Department of Radiation Oncology Osaka University Graduate School of Medicine Osaka Japan; ^3^ Osaka Heavy Ion Therapy Center Osaka Japan

**Keywords:** brain metastasis, stereotactic radiosurgery, stereotactic radiotherapy

## Abstract

**Background and Purpose:**

Few reports include volumetric measurements as endpoints after stereotactic radiotherapy (SRT) despite the importance of such measurements. This study aimed to (1) investigate the impact of the volumetric response (specifically, an over 65% and over 90% volume reduction in brain metastases) at 6 months post‐SRT on local control and (2) identify the predictive factors for a volumetric response of over 65% and over 90%.

**Materials and Methods:**

This study included 250 unresected brain metastases (>0.3 cc) treated with SRT. Doses were stratified according to the biological effective dose (BED). The BED was calculated using four models: linear‐quadratic (LQ): α/β = 10; LQ: α/β = 20; LQ cubic: α/β = 12; and LQ linear: α/β = 10. The median prescription dose was 30 Gy/3 fractions (BED20, 45). The median follow‐up time after SRT was 18.6 months (range, 6.4–81.8 months).

**Results:**

In the multivariate analysis, over 65% volume reduction and over 90% volume reduction were prognostic factors for local control (hazard ratio: 2.370, *p* = 0.011 and hazard ratio: 3.161, *p* = 0.014, respectively). A dose of 80% of the gross tumor volume (GTV) D80 > BED20 58 was a predictive factor for over 65% and over 90% volume reductions (odds ratio: 1.975, *p* = 0.023; odds ratio: 3.204, *p* < 0.001, respectively).

**Conclusion:**

Robust volume reduction of brain metastases at 6 months post‐SRT can predict local control. GTV D80 in the LQ model: α/β = 20 may be warranted for good volume reduction.

## Graphical abstract

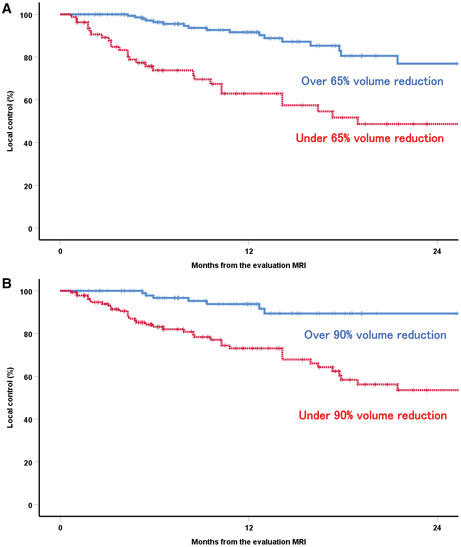

## 1 INTRODUCTION

Stereotactic radiotherapy (SRT) is one of the most important treatment modes for brain metastases.^1^, ^2^ It includes both stereotactic radiosurgery and fractionated stereotactic radiotherapy.^1^, ^2^, ^3^ The mass effect of brain metastases and peritumoral edema often causes severe neurological symptoms that worsen the quality of life (QOL).^4^, ^5^ Reducing the volume of brain metastases reduces the amount of peritumoral edema and leads to fewer neurological symptoms and improved QOL.^4^, ^5^ Early volumetric reduction after SRT is a prognostic factor for local tumor control.^1^, ^5^

The criteria used to assess response and progression in brain metastases are heterogeneous.^6^, ^7^ The Response Assessment in Neuro‐Oncology Brain Metastases (RANO‐BM) guidelines are the first step toward standardizing the criteria.^8^ These guidelines suggest including volumetric reduction as a study endpoint because it is more reliable than using the sum of the longest diameter of the tumor.^9^ However, few reports include this endpoint because volumetric analysis after SRT takes time and effort.^1^, ^2^, ^5^, ^8^

Over 65% volume reduction in brain metastases corresponds to a partial response in the RANO‐BM guidelines.^8^ However, in a previous study, over 65% volume reduction at 3 months post‐SRT did not predict local control.^1^ Whether over 65% volume reduction at 6 months post‐SRT would do so has yet to be investigated. It is possible that a reduction of over 90% is necessary for the achievement of local control.

This study aimed to (1) investigate the effect of the volumetric response (specifically, over 65% and over 90%) at 6 months post‐SRT on local control and (2) identify the predictive factors for a volumetric response of over 65% and over 90%.

## 2 MATERIALS AND METHODS

### 2.1 Patients

This study included 147 patients with 250 unresected brain metastases treated with SRT at the Osaka International Cancer Institute between 2013 and 2020, identified from our electronic database. The exclusion criteria were as follows: no magnetic resonance imaging (MRI) 5.0–8.5 months after SRT, whole‐brain radiotherapy before the evaluation MRI, brain metastases with local tumor progression before the evaluation MRI, and brain metastases <0.3 cc at baseline. The evaluation MRI was the MRI performed nearest to 6 months (median, 6.3 months; range, 5.0–8.5 months) after SRT.

All patients provided written informed consent for the use of their data prior to starting SRT. The institutional review of the board of the Osaka International Cancer Institute approved this study (21150). Table 1 lists the characteristics of the patients and their brain metastases. A total of 107 patients received systemic therapy concurrently (within 1 month before or after SRT). Of 107 patients, 30 patients received immunotherapy including immune checkpoint inhibitors and 58 patients received target therapy including angiogenesis inhibitors, human epidermal growth factor receptor type 2 targeted agents, tyrosine kinase inhibitors, and cyclin‐dependent kinase inhibitors.

**TABLE 1:** Patient, brain metastases, and SRT characteristics

Characteristic	Category	Value	
Patients (*n* = 147)			
Age (years)	Median (range)	66	(22–85)
	22–65, *n* (%)	70	(47.6)
	>65, *n* (%)	77	(52.4)
Sex, *n* (%)	Male	73	(49.7)
	Female	74	(50.3)
PS, *n* (%)	0	95	(64.6)
	1	35	(23.8)
	2	13	(8.8)
	3	4	(2.7)
Primary cancer, *n* (%)	Lung	97	(66.0)
	Breast	19	(12.9)
	GI	8	(5.4)
	Kidney	7	(4.7)
	Melanoma	5	(3.4)
	Others	11	(7.5)
Brain metastases (*n* = 250) and SRT			
PTV prescription dose, *n* (%)	20 Gy/1 fraction	15	(6.0)
	24 Gy/1 fraction	90	(36.0)
	30 Gy/3 fractions	48	(19.2)
	30 Gy/5 fractions	3	(1.2)
	35 Gy/5 fractions	94	(37.6)
GTV (cc)	Median (range)	1.1	(0.3–33.1)
	0.3–1, *n* (%)	118	(47.2)
	1–4, *n* (%)	95	(38.0)
	>4, *n* (%)	37	(14.8)
SRT modality, *n* (%)	Conformal RT	12	(4.8)
	Manual VMAT	103	(41.2)
	HA VMAT	135	(54.0)

Abbreviations: GI, gastrointestinal; GTV, gross tumor volume; HA VMAT, HyperArc volumetric modulated arc therapy; PS, performance status; PTV, planning target volume; RT, radiotherapy; SRT, stereotactic radiotherapy.

### 2.2 Treatments

The SRT treatment has been previously described.^10^, ^11^ For simulation, the patient was immobilized using a thermoplastic mask, and planning computed tomography (CT) was performed with a thickness of 1 mm. Planning CT scans were loaded into a treatment planning system (Eclipse; Varian Medical Systems, Palo Alto, CA, USA). The gross tumor volume (GTV) was delineated by referring to a T1‐weighted, gadolinium‐enhanced magnetic resonance image. The planning target volume (PTV) was determined by adding an isotropic margin of 1 mm (range, 1–3 mm) to the GTV.

The median prescription dose was 30 Gy/3 fractions to cover 95% of the volume of the combined PTV. The median isodose (prescription dose/max dose × 100) was 79.2% (range, 43.0–92.8%). The isodose was determined by the physician's preference, and dose inhomogeneity was allowed within the GTV. From 2013 to 2019, we ordinarily prescribed 30 Gy/3 fr or 30–35 Gy/5 fr for GTV >4 cc and 20–24 Gy/1 fr for GTV <4 cc. From 2020, we ordinarily prescribed 35 Gy/5 fr in any case. Doses to the brain tissue were reduced to the minimum in the optimization process. All treatments were performed using a C‐arm linear accelerator (Linac) (Clinac 23Ex, Ture Beam STX, or Edge; Varian Medical Systems, Palo Alto, CA, USA).

Follow‐ups included clinical examination and MRI and were performed at least every 4 months during the first 2 years after SRT initiation and at least every 6 months afterward. The interval was shortened when the tumor volume increased or new symptoms developed. The median follow‐up time after SRT initiation was 18.6 months (range, 6.4–81.8 months).

The evaluation MRI was loaded into the treatment planning system and registered with the SRT plan. The radiation oncologist delineated the tumor after referring to the T1‐weighted, gadolinium‐enhanced MRI. Tumor contouring on evaluation MRI included the treatment effects. The volume of the tumor (cc) on evaluation MRI was evaluated, and the volume reduction rate from GTV (cc) was calculated.

### 2.3 Definitions

Local control was calculated from the time of the evaluation MRI to the radiological observation of tumor progression of a treated lesion. Tumor progression was defined according to the RANO‐BM guidelines.^8^ When a differential diagnosis of tumor progression and brain necrosis was needed, tumor progression was defined as the correspondence between the contrast‐enhanced volume on the T1‐weighted MRI scans and the low signal‐defined lesion margin on the T2‐weighted MRI scans^12^ and/or if the maximum standardized uptake value (SUV) within the tumor/SUV within the normal gray matter was over 1.4 on ^11^C‐methionine positron emission tomography.^13^

### 2.4 Statistical analysis

Local control rates and hazard ratios (HRs) were estimated using the Kaplan–Meier method and Cox proportional hazard models, respectively. A univariate analysis using a logistic regression model was performed to determine odds ratios (ORs) for a set of candidate predictor variables; this analysis showed the raw uncorrected effects of each variable for over 65% and over 90% volume reduction.

For the GTV parameter, GTV D100, D98, D80, D60, D40, D20, D2, and Dmax were analyzed. Doses were stratified according to the biological effective dose (BED). It is a matter of debate which formula should be used to calculate the BED for SRT for brain metastases^2^, ^14^, ^15^, ^16^, ^17^, ^18^; hence, we used four models: two linear‐quadratic (LQ) models: α/β = 10 and α/β = 20; the linear‐quadratic cubic (LQC) model: α/β = 12; and the linear‐quadratic linear (LQL) model: α/β = 10. The formulas for these models are as follows:

BED LQ models = nd [1 + d/(α/β)].

BED LQC model = nd [1 + d/(α/β) ‐ d^2^/(α/γ)].

BED LQL model = nd [1 + d*G(δd)/(α/β)].

where n is the number of fractions, d is the dose per fraction, α/γ is 648 Gy^2^, δ is 0.14, and G(^χ^) = (2/^χ2^)(^χ^‐1+ *e* ^‐χ^).^2^, ^14^, ^15^, ^16^, ^17^

In the univariate analysis, we used the Pearson correlation coefficient (r) to evaluate the correlation between the GTV parameters themselves, and we used the Spearman's correlation coefficient to evaluate the correlation between the clinical (treatment) parameters and the GTV parameters. When either coefficient for the candidate prognostic factors was >0.40 in the univariate analysis, we selected only one variable for the multivariate analysis. The GTV parameter with the lowest Akaike information criterion (AICc) value was deemed the most predictive. Evidence ratios (EVRs) were calculated; models with an EVR <2.7 were considered to have substantial support.^19^ Statistical significance was set at a *p*‐value of <0.05. Statistical analysis was performed using SPSS version 25 software (SPSS Inc., Chicago, IL, USA).

## 3 RESULTS

### 3.1 Local control

**FIGURE 1:**
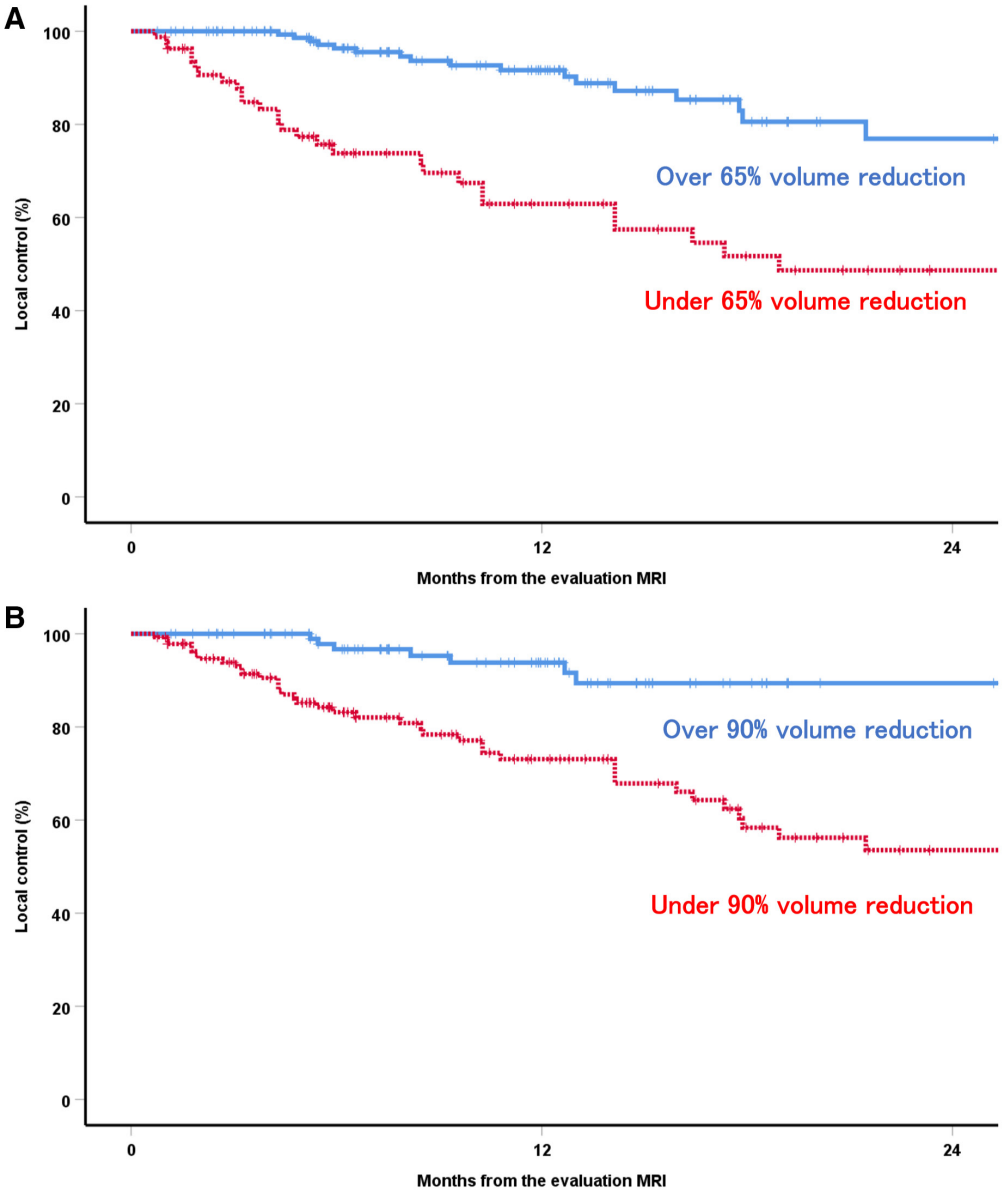
Comparison of the local control rates for over vs under 65% volume reduction (A) and over 90% volume reduction (B) at the evaluation MRI. MRI, magnetic resonance imaging.

Over 65% and over 90% volume reduction approximately 6 months post‐SRT (i.e., at the evaluation MRI) was achieved for 169 (67.6%) and 111 (44.4%) of the 250 brain metastases, respectively. The overall local control rate at 0.5 and 1.5 years from the evaluation MRI (approximately 1 and 2 years after SRT, respectively) was 89.4% and 71.3%, respectively. The local control rate at 0.5 years from the evaluation MRI was 96.3% vs 73.8% for over vs under 65% volume reduction and 96.7% vs 83.2% for over vs under 90% volume reduction (Figure 1).

The results of the univariate and multivariate analyses for local control are shown in Table 2. Over 65% and over 90% volume reduction were prognostic factors for local control in the multivariate analysis (HR: 2.370, *p* = 0.011 and HR: 3.161, *p* = 0.014, respectively).

**TABLE 2:** Results of the univariate and multivariate analyses of potential prognostic factors for local control

Variable	Univariate		Multivariate	
	HR (95% CI)	*p*‐value	HR (95% CI)	*p*‐value
>65% volume reduction at the evaluation MRI				
Yes	1	<0.001	1	0.011
No	3.897 (2.181–6.962)		2.370 (1.219–4.607)	
>90% volume reduction at the evaluation MRI				
Yes	1	<0.001	1	0.014
No	5.649 (2.533–12.596)		3.161 (1.259–7.940)	
GTV (cc)				
0.3–1	1	<0.001	1	<0.001
>1	5.657 (2.534–2.630)		5.685 (2.542–12.716)	
Age (years)				
22–65	1	0.194		
>65	1.461 (0.824–2.589)			
PS				
0–1	1	0.650		
2–3	0.788 (0.281–2.207)			
Primary cancer				
Lung, breast	1	0.580		
Others	0.812 (0.388–1.699)			
# of fractions				
1 (SRS)	1	0.323		
>1 (FSRT)	1.347 (0.746–2.430)			
SRT modality				
HA VMAT	1	0.674		
Others	1.136 (0.627–2.061)			

Abbreviations: CI, confidence interval; FSRT, fractionated stereotactic radiotherapy; GTV, gross tumor volume; HA VMAT, HyperArc volumetric modulated arc therapy; HR, hazard ratio; MRI, magnetic resonance imaging; PS, performance status; SRS, stereotactic radiosurgery; SRT, stereotactic radiotherapy.

### 3.2 Analysis of over 65% and 90% volume reduction at 6 months post‐SRT

Over 65% volume reduction significantly correlated with improved performance status in the univariate analysis (Table 3). GTV D80 in the LQ model: α/β = 20 had the lowest AICc value for both over 65% and over 90% volume reduction (Table 4). This model was the only model with an EVR of <2.7 for over 65% volume reduction, while GTV D80 and D98 in the LQ model: α/β = 20 was the only model with an EVR of <2.7 for over 95% volume reduction (Table 4).

**TABLE 3:** Results of the univariate analysis for prognostic factors for over 65% and over 90% volume reduction at the evaluation MRI

Variable	Over 65%		Over 90%	
	OR (95% CI)	*p*‐value	OR (95% CI)	*p‐*value
Age (years)				
22–65	1	0.450	1	0.488
>65	0.815 (0.480–1.385)		0.838 (0.508–0.381)	
PS				
0–1	1	0.009	1	0.052
2–3	0.367 (0.173–0.779)		0.445 (0.197–1.006)	
Primary cancer				
Lung, breast	1	0.141	1	0.220
Others	0.626 (0.336–1.168)		0.679 (0.365–1.261)	
# of fractions				
1 (SRS)	1	0.788	1	0.922
>1 (FSRT)	1.076 (0.630–1.839)		0.975 (0.588–1.616)	
SRT modality				
HA VMAT	1	0.377	1	0.810
Others	1.272 (0.746–2.172)		1.063 (0.645–1.754)	
GTV (cc)				
0.3–1	1	0.546	1	0.240
>1	0.849 (0.499–1.445)		0.741 (0.449–1.222)	
Time of the Evaluation MRI (months)				
5–6.5	1	0.181	1	0.263
6.5–8.5	1.443 (0.843–2.472)		1.332 (0.806–2.199)	
Each GTV dose for BED LQ model: α/β = 10				
D100	1.010 (0.992–1.029)	0.286	1.012 (0.994–1.030)	0.193
D98	1.018 (0.998–1.039)	0.084	1.017 (0.997–1.036)	0.089
D80	1.026 (1.005–1.048)	0.015	1.022 (1.002–1.042)	0.028
D60	1.020 (1.002–1.038)	0.033	1.016 (1.000–1.033)	0.056
D40	1.023 (1.006–1.039)	0.007	1.018 (1.003–1.034)	0.019
D20	1.018 (1.005–1.031)	0.008	1.014 (1.002–1.026)	0.021
D2	1.014 (1.003–1.024)	0.012	1.012 (1.002–1.021)	0.016
Dmax	1.013 (1.003–1.023)	0.013	1.011 (1.002–1.020)	0.018
Each GTV dose for BED LQ model: α/β = 20				
D100	1.037 (0.997–1.080)	0.071	1.040 (1.000–1.082)	0.050
D98	1.062 (1.017–1.109)	0.007	1.056 (1.011–1.102)	0.013
D80	1.067 (1.025–1.110)	0.001	1.054 (1.016–1.094)	0.006
D60	1.041 (1.010–1.073)	0.010	1.032 (1.004–1.061)	0.027
D40	1.037 1.011–1.064)	0.005	1.028 (1.005–1.051)	0.017
D20	1.026 (1.007–1.046)	0.009	1.020 (1.002–1.037)	0.025
D2	1.019 (1.004–1.035)	0.016	1.015 (1.002–1.029)	0.023
Dmax	1.018 (1.003–1.032)	0.018	1.014 (1.002–1.027)	0.025
Each GTV dose for BED LQC model: α/β = 12				
D100	1.042 (0.998–1.088)	0.063	1.040 (0.996–1.085)	0.073
D98	1.050 (1.008–1.094)	0.019	1.038 (0.999–1.078)	0.054
D80	1.040 (1.007–1.073)	0.016	1.028 (0.999–1.057)	0.056
D60	1.027 (1.002–1.053)	0.031	1.019 (0.997–1.041)	0.092
D40	1.021 (1.001–1.041)	0.038	1.015 (0.997–1.032)	0.095
D20	1.016 (1.000–1.031)	0.045	1.011 (0.998–1.025)	0.100
D2	1.012 (1.000–1.024)	0.060	1.009 (0.999–1.020)	0.082
Dmax	1.011 (0.999–1.022)	0.063	1.009 (0.999–1.019)	0.083
Each GTV dose for BED LQL model: α/β = 10				
D100	1.041 (0.999–1.086)	0.056	1.037 (0.997–1.080)	0.072
D98	1.046 (1.007–1.087)	0.020	1.035 (0.999–1.072)	0.058
D80	1.038 (1.007–1.069)	0.016	1.027 (0.999–1.055)	0.057
D60	1.026 (1.002–1.051)	0.034	1.018 (0.997–1.040)	0.096
D40	1.021 (1.002–1.041)	0.032	1.015 (0.998–1.033)	0.087
D20	1.016 (1.001–1.032)	0.038	1.012 (0.998–1.026)	0.090
D2	1.013 (1.000–1.025)	0.050	1.010 (0.999–1.021)	0.074
Dmax	1.012 (1.000–1.024)	0.053	1.010 (0.999–1.020)	0.074
Each GTV dose for BED LQ model: α/β = 20				
D80 < BED 58	1	0.007	1	<0.001
D80 > BED 58	2.162 (1.237–3.776)		3.356 (1.861–6.050)	

*Note*: For BED variables OR, increase per 1.

**TABLE 4:** AICc and EVR values for over 65% and over 95% volume reduction at the evaluation MRI

Model	GTV dose	Over 65%		Over 90%	
		AICc	EVR	AICc	EVR
BED LQ: α/β = 10	D100	315.797	125.721	343.734	23.976
	D98	313.906	48.841	342.520	13.067
	D80	310.811	10.392	340.508	4.778
	D60	312.180	20.606	341.657	8.487
	D40	309.301	4.884	339.752	3.274
	D20	309.599	5.669	340.000	3.706
	D2	310.188	7.611	339.536	2.939
	Dmax	310.393	8.432	339.644	3.102
BED LQ: α/β = 20	D100	313.690	43.841	341.444	7.630
	D98	309.484	5.353	338.983	2.229
	D80	306.129	1.000	337.380	1.000
	D60	309.909	6.620	340.392	4.509
	D40	308.419	3.143	339.574	2.995
	D20	309.595	5.658	340.341	4.395
	D2	310.636	9.522	340.197	4.090
	Dmax	310.869	10.698	340.307	4.321
BED LQC: α/β = 12	D100	313.444	38.767	342.119	10.693
	D98	311.240	12.879	341.659	8.496
	D80	310.816	10.418	341.746	8.873
	D60	312.031	19.126	342.591	13.539
	D40	312.374	22.705	342.636	13.847
	D20	312.708	26.831	342.724	14.470
	D2	313.193	34.194	342.400	12.306
	Dmax	313.298	36.038	342.418	12.417
BED LQL: α/β = 10	D100	313.218	34.625	342.112	10.655
	D98	309.355	5.018	341.760	8.935
	D80	310.893	10.827	341.766	8.963
	D60	312.237	21.201	342.650	13.944
	D40	312.110	19.897	342.483	12.827
	D20	312.400	23.002	342.556	13.304
	D2	312.878	29.212	342.224	11.269
	Dmax	312.981	30.755	342.235	11.331

Abbreviations: AICc, Akaike Information Criterion; BED, biological effective dose; EVR, evidence ratio; GTV, gross tumor volume; LQ, linear‐quadratic; LQC, linear‐quadratic cubic; LQL, linear‐quadratic linear; MRI, magnetic resonance imaging.

GTV D80 > BED20 58 predicted over 65% and over 90% volume reduction at 6 months post‐SRT in the univariate analysis (Table 3). For GTV D80 > BED20 58, over 65% and 90% volume reductions were achieved in 73.1% and 53.2% (vs 55.7% and 25.3% for GTV D80 < BED20 58) of the brain metastases, respectively. GTV D80 > BED20 58 was also a predictive factor for over 65% and over 90% volume reduction in the multivariate analysis (OR: 1.975, *p* = 0.023 and OR: 3.204, *p* < 0.001, respectively) (Table 5).

**TABLE 5:** Results of the multivariate analysis for prognostic factors for over 65% and 90% volume reduction at the evaluation MRI

Variable	Over 65%		Over 90%	
	OR (95% CI)	*p*‐value	OR (95% CI)	*p*‐value
Each GTV dose for BED LQ model: α/β = 20				
D80 < BED 58	1	0.023	1	<0.001
D80 > BED 58	1.975 (1.096–3.557)		3.204 (1.735–5.918)	
PS				
0–1	1	0.063	1	0.369
2–3	0.474 (0.216–1.042)		0.674 (0.284–1.596)	
Primary cancer				
Lung, breast	1	0.157	1	0.298
Others	0.630 (0.333–1.195)		0.710 (0.373–1.353)	
Each GTV (cc)				
0.3–1	1	0.870	1	0.580
>1	0.955 (0.548–1.664)		0.861 (0.508–1.460)	
Time of the evaluation MRI				
5–6.5 months	1	0.225	1	0.208
6.5–8.5 months	1.417 (0.807–2.487)		1.407 (0.827–2.396)	

Abbreviations: BED, biological effective dose; CI, confidence interval; FSRT, fractionated stereotactic radiotherapy; GTV, gross tumor volume; HA VMAT, HyperArc volumetric modulated arc therapy; LQ, linear‐quadratic; LQC, linear‐quadratic cubic; LQL, linear‐quadratic linear; MRI, magnetic resonance imaging; OR, odds ratio; PS, performance status; SRS, stereotactic radiosurgery.

## 4 DISCUSSION

This is the first study to report that over 65% and over 90% volume reduction at 6 months post‐SRT were prognostic factors for local control. Our study results agree with similar studies.^1^, ^4^, ^5^ In a previous study, an over 20% volume reduction at 3 months post‐SRT predicted local control.^1^ However, an over 65% volume reduction at 3 months post‐SRT did not predict local control.^1^ An over 65% volume reduction in brain metastases corresponds to a partial response in the RANO‐BM guidelines.^8^ Three months post‐SRT seems early for evaluation, and 6 months post‐SRT may be adequate for evaluation according to the definition of RANO‐BM guidelines of partial response.^8^ Maximum volume reduction of brain metastases leads not only to long‐term local control but may also improve peritumoral edema and QOL.^4^ Consequently, the patient may be able to receive and endure systemic therapy.^4^ Because systemic therapy is advancing, maximum volume reduction of brain metastases is becoming increasingly important these days.

The GTV parameters that correlated with over 65% as well as over 90% volume reduction at 6 months post‐SRT were investigated. GTV D80 in the LQ model: α/β = 20 best predicted both, and thus, is apparently important for favorable volume reduction after SRT. GTV D80 > BED20 58 also predicted over 65% and over 90% volume reductions. When the prescribed dose is 30 Gy/3 fractions (the median dose in this study) in an 80% isodose, achieving GTV D80 > BED20 58 is difficult; an inhomogeneous dose distribution is required. In previous studies, inhomogeneous dose distribution correlated with good local control after Gamma Knife radiotherapy^20^, ^21^ and resulted in better local control than did homogeneous distribution after Linac‐based SRT.^22^ BED20 58 is approximately 41.5 Gy/5 fractions and is similar to BED10 80. Matsuyama et al. reported that BED10 80 for the PTV predicted good local control after SRT for brain metastases.^23^ Hence, long‐term local control requires a relatively high dose. Based on these results, we added constraints to the GTV dose in our protocol in 2022. In our new protocol, the prescription dose for the PTV (GTV + 1 mm margin) was 35 Gy/5 fractions (BED20 47), which resulted in a more inhomogeneous dose distribution. For GTV >0.3 cc, GTV D80 > 50 Gy/5 fractions (BED20 75) was used when possible.

It is highly debated which of the following models should be chosen when performing SRT for brain metastases: LQ: α/β = 10; LQ: α/β = 20; LQC: α/β = 12; or LQL: α/β = 10.^2^, ^14^, ^15^, ^16^, ^17^, ^18^ LQ: α/β = 10 is well suited for stereotactic body radiotherapy for early‐stage lung cancer.^14^ However, it is not appropriate for late‐stage lung cancer with brain metastases, which are best treated using LQL: α/β = 10.^14^ The HyTEC group reported that LQ: α/β = 20 better predicted local control than LQ: α/β = 10 in patients with brain metastases.^18^ In the present study, GTV D80 in the LQ model: α/β = 20 predicted over 65% and over 90% volume reduction at 6 months post‐SRT. Various doses and fractions are used in SRT and need to be stratified according to the BED in the future. More studies are needed to determine which model best predicts local control and volume reduction after SRT for brain metastases.

When treating brain metastases via SRT, each GTV is often very small, and the median GTV is often approximately 0.2 cc.^1^, ^24^, ^25^ In a previous study, GTV of >0.2 cc was a risk factor for local recurrence; when the GTV was <0.2 cc, very good local control was obtained after treatment with an 80% isodose line.^25^ In very small brain metastases, a homogenous distribution is sufficient for tumor control. Our study excluded brain metastases with a GTV of <0.3 cc because they are not suitable for volumetric measurements after SRT. An inhomogeneous distribution may be more important when the GTV volume was over 0.3 cc.

There were some limitations to our study. First, because it was retrospective, the timing of the evaluation imaging was somewhat variable. The median time for the evaluation MRI was 6.3 months, with a range of 5–8.5 months. However, the time of the evaluation MRI (5–6.5 vs 6.5–8.5 months post‐SRT) was not a predictive factor for an over 65% and 90% response. In a previous study, the median volume reduction rate was 44.2% at 3 months, 69.6% at 6 months, and 75.3% at 12 months after SRT.^1^ Tumor volume changes dramatically within the first 3 months after SRT.^1^ Hence, our finding that over 65% volume reduction at 6 months post‐SRT predicts local control is reasonable. The volumetric analysis takes time and effort, and even retrospective reports of volumetric measurements are limited.^1^, ^2^, ^5^, ^8^ More retrospective studies that include volumetric analysis are needed. Second, our study included a variety of primary cancers, and the effects of systemic therapy were not analyzed. Systemic therapy is rapidly advancing and may be influencing tumor response. More studies of the impact of systemic therapy on brain metastases are required.

In conclusion, over 65% and over 90% volume reduction of brain metastases at 6 months post‐SRT predicts good local control. Beneficial volume reduction may require increasing the dose to GTV D80 in the LQ model: α/β = 20, and inhomogeneous dose distribution may be required for SRT for brain metastases. Further studies are needed to confirm these findings.

## CONFLICT OF INTEREST

The authors declare no conflict of interest.

## AUTHOR CONTRIBUTIONS

Conception and design: Naoyuki Kanayama, Toshiki Ikawa, Takero Hirata. Acquisition of data, analysis, and/or interpretation of data: Naoyuki Kanayama, Toshiki Ikawa, Shingo Ohira, Takero Hirata, Masahiro Morimoto, Teruki Teshima, Koji Konishi. Manuscript writing and final approval of manuscript: All authors.

## ETHICAL APPROVAL STATEMENT

The institutional review of the board of the Osaka International Cancer Institute approved this study (21150).

## PATIENT CONSENT STATEMENT

All patients provided written informed consent for the use of their data prior to starting SRT.

## Data Availability

The data that support the findings of this study are available on request from the corresponding author. The data are not publicly available due to privacy or ethical restrictions.
